# Effects of a 1-mm difference in bearing thickness on intraoperative bearing movement and kinematics in Oxford unicompartmental knee arthroplasty

**DOI:** 10.1186/s12891-022-05203-1

**Published:** 2022-04-09

**Authors:** Kohei Kawaguchi, Hiroshi Inui, Shuji Taketomi, Ryota Yamagami, Kenichi Kono, Shin Sameshima, Tomofumi Kage, Sakae Tanaka

**Affiliations:** Department of Orthopaedic Surgery, Faculty of Medicine, The University of Tokyo, 7-3-1 Hongo, Bunkyo-ku, Tokyo, 113-0033 Japan

**Keywords:** Unicompartmental knee arthroplasty, Mobile bearing, Kinematics, Intraoperative movement, Navigation system

## Abstract

**Background:**

The choice of mobile bearing (MB) thickness is essential for obtaining successful results after mobile-bearing Oxford unicompartmental knee arthroplasty (UKA). This study aimed to investigate the effects of a 1-mm difference in bearing thickness on intraoperative MB movement and intraoperative knee kinematics in Oxford UKAs.

**Methods:**

We prospectively investigated the effects of a 1-mm difference in bearing thickness on intraoperative MB movement and knee kinematics in 25 patients who underwent Oxford UKAs when surgeons didn’t know which bearing thickness to choose with 1-mm difference. A trial tibial component that was scaled every 2 mm was used to measure the intraoperative MB movement, and the tibial internal rotation relative to the femur and the knee varus angle was simultaneously evaluated using the navigation system as the knee kinematics. We separately evaluated sets of two MB thicknesses with 1-mm differences, and we compared the intraoperative parameters at maximum extension; 30º, 45º, 60º, and 90º flexion; and maximum flexion between the thicker MB (thick group) and the thinner MB (thin group).

**Results:**

The MB in the thin group was located significantly posteriorly at 90º flexion compared with that in the thick group; however, there were no differences at the other flexion angles. There was significantly less tibial internal rotation in the thin group at 90º flexion than that in the thick group; however, there were no differences at the other flexion angles. The knee varus angles in the thick group were significantly smaller than those in the thin group by approximately one degree at all angles other than at 30º and 45º flexion.

**Conclusion:**

The thicker MB could bring the less posterior MB movement and the more tibial internal rotation at 90º flexion, additionally the valgus correction angle in the thicker MB should be paid attention. These results could help surgeons to decide the thickness of MBs when they wonder the thickness of MB.

## Background

The mobile bearing (MB) Oxford unicompartmental knee arthroplasty (UKA) (Biomet Ltd., Swindon, United Kingdom) procedure has been successfully performed for more than 40 years to treat anteromedial osteoarthritis or osteonecrosis of the knee [1, 2]. The MB Oxford UKA has some advantages including a low rate of bearing wear, favorable longevity, and minimized shear stress at the bone-implant interfaces [1, 3, 4]. These advantages come from the features of the MB. However, given its mobile mechanism, there is concern that bearing dislocation can occur in 0–5.3% of all cases [1, 2, 5–7], and such dislocation occurs more frequently in Asian patients than in Western populations because of the former’s traditional lifestyle and religious behavior, which involves deep knee flexion or cross-legged sitting [5, 6]. However, to avoid bearing dislocation due to a thin MB, a thicker MB may be used, which could induce the progression of lateral compartment osteoarthritis, and lateral osteoarthritis progression is one of major reasons for revision surgery in UKA [8]. Therefore, we believed that determining the optimal MB thickness was very important when performing Oxford UKAs.

Recently, some studies have focused on intraoperative MB movement and revealed its tendencies [9, 10]. Analyzing MB movement may be worthwhile to prevent bearing dislocation. Intraoperative knee kinematics (tibiofemoral rotation, varus/valgus, etc.) provided by the navigation system have been validated as an important factor affecting clinical results in total knee arthroplasty [11, 12], and they have recently been reported as predictors of postoperative clinical outcomes in UKA [13, 14]. However, the effect of the bearing thickness on intraoperative MB movement and knee kinematics has not been reported previously. Surgeons often wonder which of two MBs with a 1-mm difference is better intraoperatively. Therefore, this study aimed to prospectively investigate the effects of a 1-mm difference in bearing thickness on intraoperative MB movement and intraoperative knee kinematics in Oxford UKAs. We hypothesized that a thicker bearing could decrease the intraoperative bearing movement and could alter the intraoperative knee kinematics in Oxford UKAs.

## Materials and methods

**Fig. 1:**
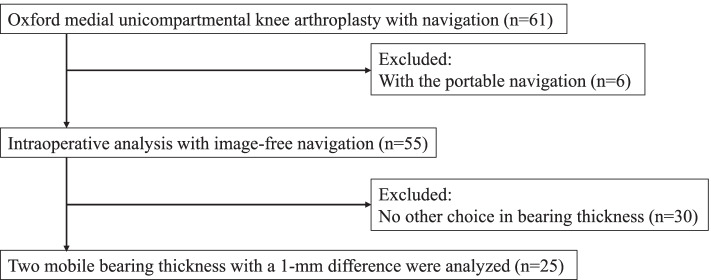
Flowchart of patient selection

Patients who underwent an Oxford medial UKA for unilateral isolated medial osteoarthritis or medial osteonecrosis between December 2017 and December 2020 were recruited. Patients who underwent a UKA with portable navigation were excluded from this study, and patients in whom the image-free navigation system (Precision N; Stryker Orthopedics, Mahwah, NJ) was used were prospectively included in this study. In addition, patients whose surgeons had no other choice in selecting the thickness of MB could be used were excluded from this study because we were unable to evaluate two different MB thicknesses with a 1-mm difference, which were defined as the thin and thick bearings. The study inclusion flow chart is shown in Fig. 1. Finally, 25 patients were included in this study. This study was approved by the institutional review boards of our institute (No. 2674). The patients and their families were informed that the data from their cases would be submitted for publication, and all patients provided written informed consent.

### Surgical procedure and evaluation of two MB thicknesses with a 1-mm difference

All UKAs were performed using a minimally invasive approach to comply with the Oxford Group methods [3] and, our method was previously reported [10, 13]. The surgeries were performed by six knee surgeons, and a highly experienced surgeon (HI) participated in all procedures as either the chief surgeon or first assistant. A tibial vertical cut was made at the medial edge of the anterior cruciate ligament insertion on the tibia with the sagittal saw blade aimed toward the hip center. A horizontal cut was then made using the tibial saw guide, which had a 7° built-in posterior slope set parallel to the long axis of the tibia in the coronal and sagittal planes. Femoral drilling was performed with an Oxford Microplasty device (MP: Biomet Ltd., Swindon, UK) to facilitate reproducible implant alignment [15]. After these procedures, we performed the same gap-balancing procedure between knee flexion and extension and a modified keel cutting method as that previously reported [16, 17]. With the trial components in place, we used two candidate trial MBs with a 1-mm thickness difference with the trial components to prospectively investigate the effects of a 1-mm difference in MB thickness. The combination of the two different trial MB thicknesses with a 1-mm difference ranged from 3 to 7 mm. First, we used a thinner trial MB (thin group), and the knee was manipulated through a full range of motion. We measured the intraoperative MB movement and the intraoperative kinematics using the navigation system (described below for further details). Next, we used a 1-mm thicker MB (thick group) and measured the same items in the same manner. After the trial evaluating the two bearing thicknesses, we chose the more suitable bearing thickness based on the demonstrated joint stability, total knee alignment, the security of the MB, and the absence of dislocation [18]. Finally, the tibial and femoral components were cemented, and the appropriate bearing was inserted.

### Intraoperative MB movement analysis

**Fig. 2:**
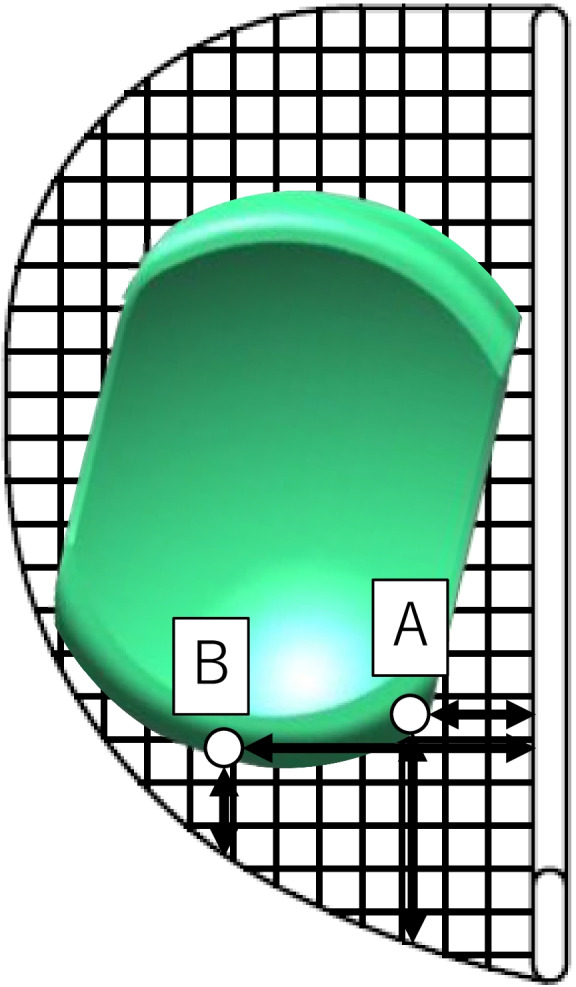
A schematic illustration of the mobile bearing and scaled trial tibial component. Point A was the front medial corner of the bearing, and Point B was the anterior midpoint of the bearing. Both of the points were evaluated as the distance from the edge of the tibial component and the distance from the lateral wall of the tibial component

The intraoperative measurement of MB movement was performed as described in a previous study [10]. A trial tibial component that was scaled every 2 mm was used to measure the intraoperative movement of the MBs (Fig. 2). After the tibial and femoral osteotomy, we set the scaled tibial component and trial femoral component. After inserting a trial MB, we evaluated bearing positions at maximum knee extension; 30°, 45°, 60°, and 90° flexion; and maximum knee flexion with the navigation system, measuring the trial bearing at two points on the scaled tibial component. Point A was located at the front medial corner of the bearing, and Point B was located at the anterior midpoint of the bearing (Fig. 2). As mentioned above, we evaluated the movement of two trial MBs (the thick and thin groups).

### Intraoperative knee kinematics analysis

**Fig. 3:**
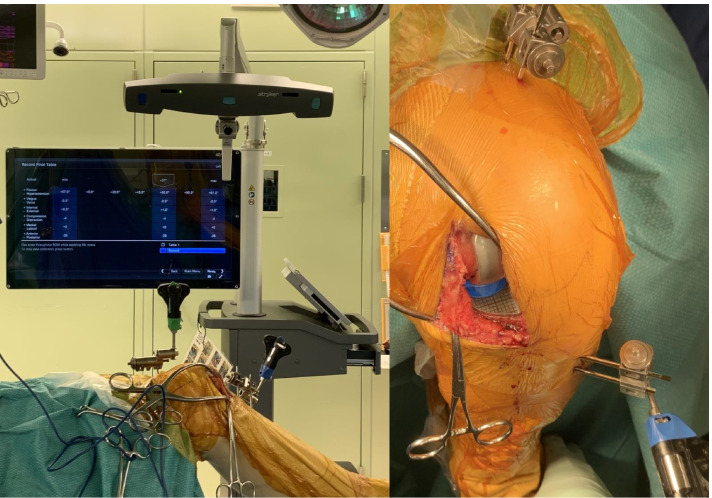
Intraoperative evaluation of knee kinematics with image-free navigation and simultaneous measurement of the intraoperative mobile bearing movement

The intraoperative tibiofemoral rotational kinematics and knee varus-valgus position during knee flexion were also evaluated using the image-free navigation system as described in a previous study [13]. After performing the osteotomy necessary for the procedure using the navigation system, we registered the anteroposterior (AP) axis of the femur and tibia to measure the rotational kinematics. The AP axis of the femur was aimed along the line connecting two peg holes, which is the rotational axis of the Oxford femoral component, and the AP axis of the tibia was aimed along the lateral wall of the tibial tray. After implanting a trial component, the tibial component internal rotation angles relative to the femoral component were evaluated in each patient using the navigation kinematic data obtained during the motion cycles, from maximum extension to maximum flexion (flexion angles at maximum extension, 30°, 60°, 90°, and maximum flexion). Tibial internal rotation was considered a positive value. Moreover, the knee varus angle (the varus angle of the tibial mechanical axis relative to the femoral mechanical axis) was measured at each knee flexion angle. As mentioned above, we evaluated two different MB thicknesses with a 1-mm difference and compared the thick and thin groups. The intraoperative knee kinematics analysis and MB movement analysis were performed simultaneously (Fig. 3).

### Statistical analysis

All statistical analyses were performed using SPSS v.25.0 statistical software (IBM Corp., Armonk, NY). Repeated measures analysis of variance (ANOVA) and post-hoc pair-wise comparison (Bonferroni test) were used to analyze differences in the intraoperative rotation angle, valgus angle, and the MB movement between the two groups. *P* values of < 0.05 were considered statistically significant for all tests. The power analysis was performed using G*Power software (version 3.1.9.2; Heinrich Heine Universität Düsseldorf, DE). A post hoc power analysis for intraoperative knee kinematics was performed, and the power calculated as 0.65.

## Results

**Fig. 4:**
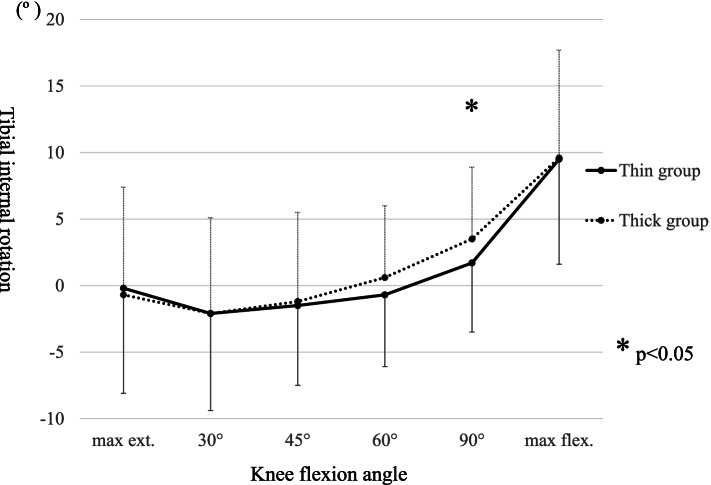
Comparison of intraoperative tibial rotation during knee flexion between the thin bearing and the thick bearing. The tibial internal rotation angle relative to the femur is a positive value. *: *P* < 0.05 significant difference. max ext.: maximum knee extension, max flex.: maximum knee flexion

**Fig. 5:**
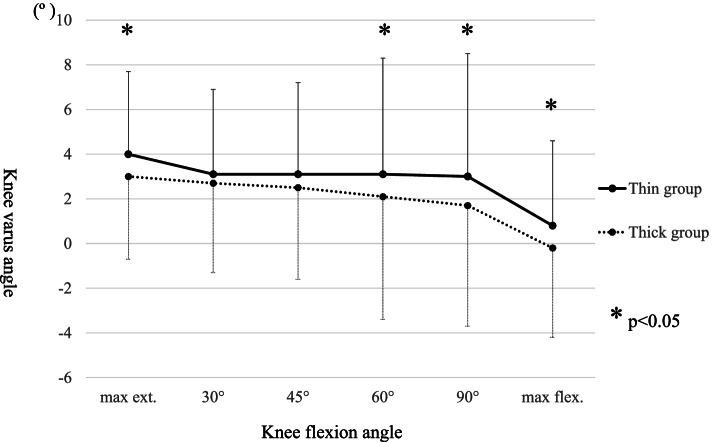
Comparison of the intraoperative knee varus angle during knee flexion between the thin bearing and the thick bearing. The varus knee angle is a positive value. max ext.: maximum knee extension, max flex.: maximum knee flexion. *: *P* < 0.05 significant difference

The baseline demographic characteristics of the enrolled patients are shown in Table 1. The intraoperative movements of Point A and Point B in the MB in both groups are shown in Table 2, respectively. The MB always moved posteriorly during knee flexion. Conversely, the MB in the thin group moved significantly more posteriorly from the anterior edge of the tibial component at 90º knee flexion than it did in the thick group; however, there was no difference in the total posterior movement of the MB from the anterior edge of the tibial component at maximum knee flexion. Additionally, there was no difference in the distance between the bearing and the lateral wall during knee flexion between the two groups. The intraoperative tibial internal rotation angle relative to the femur during knee flexion in both groups is shown in Table 3 and Fig. 4, and the tibia in the thin group was significantly internally rotated compared with that in the thick group at 90º knee flexion; however, there were no differences in the tibial internal rotation at maximum flexion and in the maximum knee angle itself between the two groups (thin group 133.0º ± 4.4º, thick group 131.9º ± 5.3º average ± standard deviation, *p* = 0.11). The intraoperative knee varus angle during knee flexion is shown in Table 3 and Fig. 5; the thick group displayed a significantly greater valgus knee angle at each knee flexion angle except for at 30º and 45º, and the difference in coronal alignment was approximately one degree. The final choice of MB thickness and the combination of the two different trial MB thicknesses is shown in Table 4.

## Discussion

There are several important findings in this study. In the thin group, the MB was located more posteriorly at 90º flexion, the tibia was less internally rotated at 90º flexion, and the knee was slightly varus during almost the almost entire range of knee flexion compared with such measurements in the thick group. However, there were no significant differences in the MB movement and the tibial internal rotation angle at maximum knee extension and flexion.

A functional normal knee has a medial pivot motion and a bicondylar rollback motion during knee flexion [19, 20], and this combination enables the knee to move comfortably and flex deeply. This study showed that the medial contact point moved posteriorly, particularly after 90º flexion, and the medial posterior movement was recognized as a bicondylar rollback movement in the entire knee kinematics. In this study, the medial MB in the thin group was located more posteriorly at 90º flexion than that in the thick group, and the tibial internal rotational angle in the thin group was smaller at 90º flexion. Therefore, if the lateral contact point similarly moved posteriorly in both groups, these results could be interpreted as a bicondylar rollback occurring earlier in the thin group and a larger medial pivot motion until 90º in the thick group. In total knee arthroplasty, medial knee stability in the mid flexion angle has been reported as an important factor resulting in better postoperative clinical outcomes, and medial pivot motion in mid flexion is also reported to be essential to successful total knee arthroplasty [12, 21]. However, there is no evidence on medial pivot motion and postoperative clinical outcomes for UKA. Additionally, there were no significant differences in the posterior MB movement and the tibial internal rotation at maximum knee flexion between the thick and thin MBs that differed by 1 mm in thickness. Therefore, the tibia in the thin group rotated internally at a deep knee flexion angle. These kinematic differences also might influence postoperative clinical results and bearing dislocation. However, in this study, we were unable to compare the postoperative clinical outcomes between the two groups because the two groups only existed intraoperatively, and we adopted the final MB from both groups. Therefore, further investigation is necessary to reveal the entire knee kinematics situation and the relationship between intraoperative kinematics and postoperative clinical outcomes in UKA. Such future studies might help determine the ideal bearing thickness when choosing between a 1-mm difference.

Postoperative bearing dislocation is one of the main reasons for revision surgery after Oxford UKA [22]; however, the precise mechanisms causing this condition remain unknown. Bae and Lewold et al. mentioned that bearing dislocation could be attributed to component malposition and soft tissue imbalance with subsequent maltracking of the meniscal bearing [23, 24]. However, Lewold did not mention what maltracking of the MB indicated in their reports [24] and Bae et al. assumed that MB posterior overhang from the posterior edge of the tibial component could induce bearing dislocation [23]. Jamshed et al. reported a 180º bearing spin motion before the posterior bearing dislocation, and they mentioned that potential bearing spin motion could occur before a bearing dislocation [25]. Therefore, the intraoperative bearing movement is important. Kawaguchi et al. reported that the component position influenced the intraoperative MB movement, and they mentioned that MBs whose femoral components were set laterally tended to move posteriorly while in contact with the lateral wall [10]. MBs that are located beside the lateral wall did not tend to spin out; therefore, the component position could be an important factor for not only the intraoperative MB movement but also the bearing dislocation. Conversely, in this study, there was no significant difference in the distance between the MB tibial lateral wall or in the bearing rotation between the two groups. Therefore, the 1-mm difference in bearing thickness did not influence the relationship between the MB and the tibial lateral wall or the bearing rotation during passive knee flexion. However, the spin out stress test and the rollover sleep test (ROS test) [18] were performed to confirm the tendency of the bearing dislocation in this study before reaching a final decision on the bearing thickness. In the spin out stress test, the bearing was manually forced to rotate internally if the bearing had a tendency to rotate over 90º. Additionally, in the ROS test, the knee was forced into the valgus position, and the femur applied stress on the medial aspect of the tibial bearing, causing elevation of the lateral edge of the bearing to confirm whether a bearing has a tendency to dislocate into the intercondylar ridge. In these procedures, there were some unacceptable cases in which bearing dislocation occurred easily in the thin group; thus, the thicker bearing was chosen as the final bearing, as shown in Table 4. In such situations, this study could be one of evidences that we could choose the thicker bearing in terms of the MB movement. In future studies, the MB movement and the knee kinematics should be evaluated in not only passive knee flexion but also in these dislocation confirming tests.

When assessing coronal alignment in Oxford UKAs, a valgus correction should be performed carefully because overcorrected coronal valgus alignment could induce the progression of arthritis on the lateral side [26], and lateral osteoarthritis progression was one of the primary reasons for revision surgery [8]. In this study, the thick group displayed a greater valgus knee angle at each knee flexion angle except for 30º and 45º; however, the difference in the valgus knee angle was an average of approximately one degree. Misir et al. revealed a difference of approximately 3.6º in the postoperative tibiofemoral angle after Oxford UKA between the lateral OA progressed group and the non-progressed group [27]; thus, the difference in this angle between the two groups was much smaller in this study than the difference in their study. Additionally, Ro et al. compared complications after Oxford Phase III UKA between Asian and Western patients and reported that although the total reoperation rates did not differ between the two populations, reoperation for bearing dislocation was more likely to occur in Asian patients than in Western patients whereas reoperation for lateral knee OA progression was more likely to occur in Western patients than in Asian patients [5]. However, overcorrection of coronal alignment after Oxford UKA should not be neglected in Asian patients. Even if the surgeon cannot determine whether to choose the thin or thick MB that differ by 1 mm, this study could give surgeons the information that the difference influence one degree in coronal alignment, even in Asian patients with varus knee deformities and this information could aid the surgeons to decide the bearing thickness.

This study has some limitations. The first limitation is that intraoperative bearing movements were evaluated with a trial MB, which differs slightly from an actual MB. An actual MB is an ‘anatomic’ bearing with an extended lateral edge. However, actual tibial components do not have a scale on the surface, and MB movement cannot be evaluated with actual MBs and tibial components. Second, the sample size was relatively small. Thus, the current findings should be confirmed in a larger cohort and the further research with a larger sample is going in our facility. Third, there could be an implantation error between the trial components and actual components. Implantation errors were checked with an intraoperative navigation system, with its alignment adjusted as little as possible. Fourth, we did not include the difference of tibial component size. Fifth, we never experienced a postoperative bearing dislocation in this series, so the reasons for bearing dislocations remain unknown. Sixth, this study included several sizes of tibial component, therefore the percentage of posterior MB movement on the tibial component might be different among tibial component sizes. Seventh, we did not distinguish between osteoarthritis and osteonecrosis.

## Conclusion

There were significant differences in the MB movement and tibial internal rotation at 90º flexion, and the knee was slightly in the varus position in the thin group. The thicker MB could bring the less posterior MB movement and the more tibial internal rotation at 90º flexion, additionally the valgus correction angle in the thicker MB should be paid attention. These results could help surgeons to decide the thickness of MBs when they wonder the thickness of MB.

**Table 1:**
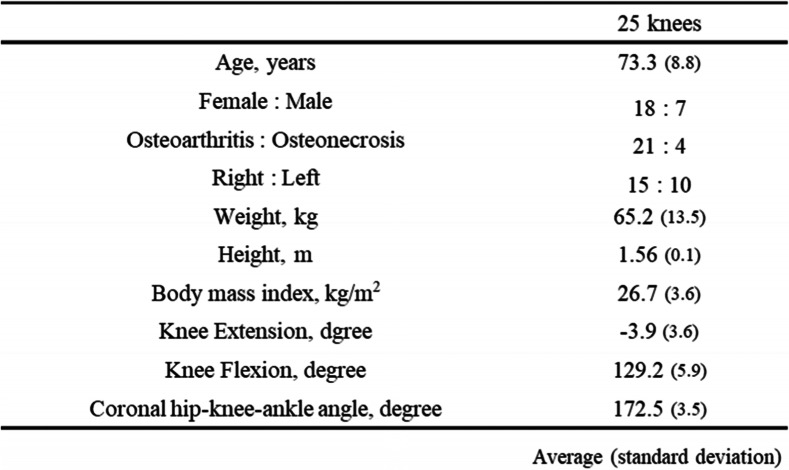
Patient demographics

**Table 2:**
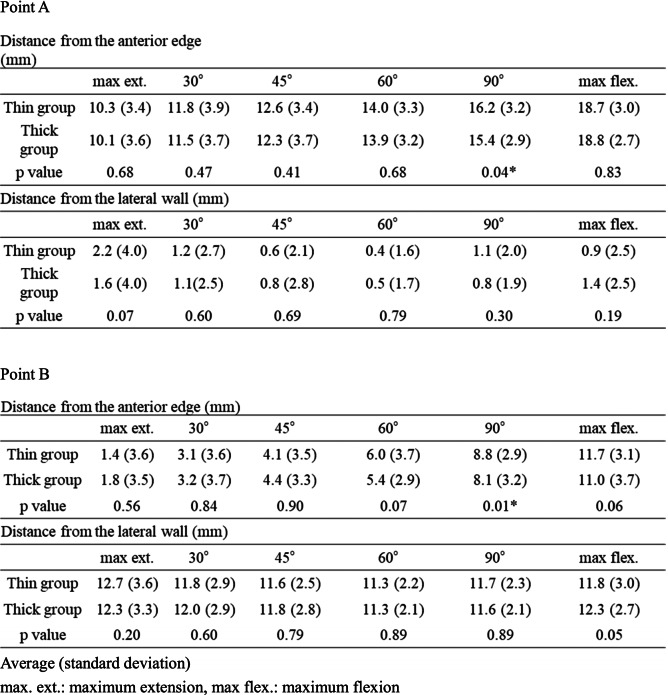
Intraoperative bearing movement of Point A and Point B for the mobile bearing

**Table 3:**
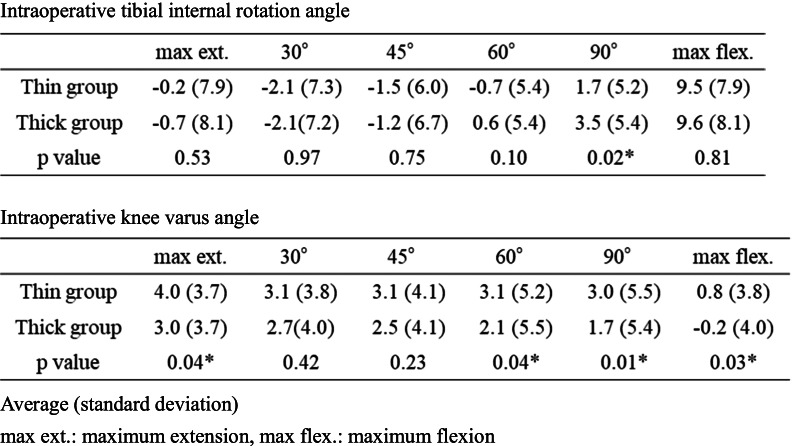
Intraoperative tibial internal rotation angle relative to the femur and Intraoperative knee varus angle

**Table 4:**
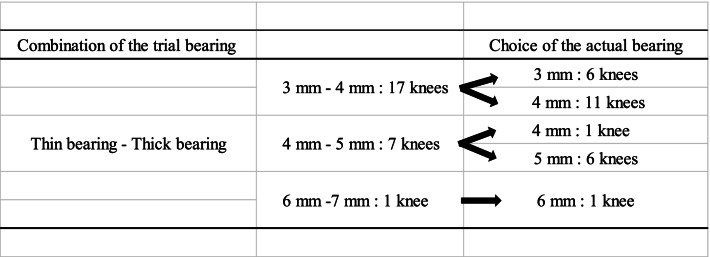
The final choice of mobile bearing thickness and the combination of the trial mobile bearing thicknesses

## Data Availability

The datasets generated and analyzed during the current study are not publicly available due to privacy concern of participants but are available from the corresponding author on reasonable request.

## Abbreviations

UKA: Unicompartmental knee arthroplasty
MB: Mobile bearing
AP: Anteroposterior
